# Application of circular statistics in temporal distribution of adult mosquitoes in Qingdao, Shandong Province, China, 2021–2023

**DOI:** 10.1186/s13071-024-06412-4

**Published:** 2024-07-30

**Authors:** Binghui Li, Qiqi Fu, Yiqing Huang, Qintong Sun, Chunchun Zhao, Xiaofang Ma, Yantao Liu

**Affiliations:** 1 Qingdao Municipal Center for Disease Control and Prevention, Qingdao, China; 2 Qingdao Institute of Preventive Medicine, Qingdao, China; 3 Shandong Provincial Center for Disease Control and Prevention, Jinan, China; 4 National Key Laboratory of Intelligent Tracking and Forecasting for Infectious Diseases, Department of Vector Biology and Control, WHO Collaborating Centre for Vector Surveillance and Management, National Institute for Communicable Disease Control and Prevention, Chinese Center for Disease Control and Prevention, Beijing, China

**Keywords:** Light trap, Mosquito, Surveillance, Seasonality, Circular statistics

## Abstract

**Background:**

Analyses of the temporal distribution of mosquitoes are presented in statistical charts, but it is difficult to prove in statistics whether differences in peak periods exist among different years or habitats. This study aimed to investigate the application of circular statistics in determining the peak period and a comparison of differences.

**Methods:**

Surveillance of adult mosquitoes was conducted twice a month by light traps in five different habitats from March to November for 3 years (2021–2023) in Qingdao, Shandong Province, China. The Kruskal–Wallis test was performed to determine the differences in mosquito density among different years and habitats. Circular statistics and line charts were employed to determine the peak period and a comparison of differences.

**Results:**

Among a total of 14,834 adult mosquitoes comprising five mosquito species from four genera, *Culex pipiens pallens* was dominant and accounted for 89.6% of the specimens identified. *Aedes albopictus*, *Armigeres subalbatus*, and *Anopheles sinensis* made up 5.7%, 4.2%, and 0.5%, respectively. *Culex tritaeniorhynchus* accounted for less than 0.1%. The mean mosquito density (females/trap night) for the trapping period was 10.3 in 2021, 5.6 in 2022, and 3.6 in 2023. Among five habitats, the highest mosquito density was 8.9 in livestock sheds, followed by 6.8 in parks, 5.9 in rural dwellings, 5.5 in urban dwellings, and 5.4 in hospitals. No statistically significant differences were found among different years (*H* = 1.96, d.f. 2, *P* = 0.376) and habitats (*H* = 0.45, d.f. 4, *P* = 0.978). Overall, the peak period of mosquito activity fell in the months from June to September. The peak period among 3 years differed significantly (*F*_(2,7022)_ = 119.17, *P* < 0.01), but there were no statistically significant differences in peak period among different habitats (*F*_(4,7020)_ = −159.09, *P* > 0.05).

**Conclusion:**

Circular statistics could be effectively combined with statistical charts to elucidate the peak period of mosquitoes and determine the differences in statistics among different years and habitats. These findings will provide valuable information for mosquito control and public health management.

**Graphical Abstract:**

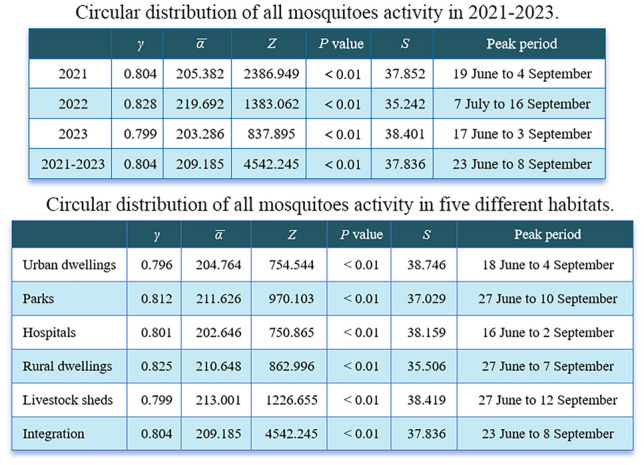

## Background

According to the World Health Organization’s “Global Vector Control Response 2017–2030” report, around 80% of the world’s population is at risk of one or more vector-borne infectious diseases (VBIDs) and about 17% of the global burden of infectious diseases is caused by VBIDs [1]. Mosquito-borne infectious diseases (MBIDs), such as malaria, filariasis, dengue fever, chikungunya fever, Japanese encephalitis, and yellow fever, pose a significant risk to both animal and human health owing to their worldwide spread [2–7]. Although the number of cases of VBIDs tends to decline with fluctuations, it still maintains a relatively high level in China [8, 9], and the epidemic area of VBIDs in China has expanded in recent years. Similarly, the incidence rate or mortality of MBIDs also showed an upward trend. In addition to the reemergence of VBIDs induced by climate change in China, exotic infectious diseases mediated by mosquito vectors are a novel threat to public health owing to the increasing levels of international/intercontinental trade and movement of people [10–13]. People who survive MBIDs can be left permanently disabled or disfigured, compounding their disadvantage. Therefore, MBIDs exact a health, social, and economic burden globally and restrict both rural and urban development.

MBIDs are transmitted to people through the bite of an infected female mosquito. *Aedes aegypti* and *Ae. albopictus* tend to be anthropophilic and are the prime vectors responsible for the transmission of several mosquito-borne viruses, including dengue virus, Zika virus, yellow fever virus, chikungunya virus, and West Nile virus [14]. These viruses exert adverse health impacts on the global population. Moreover, the main vectors of malaria caused by *Plasmodium* parasites in China include four species of *Anopheles* mosquitoes, namely *Anopheles sinensis*, *An. lesteri*, *An. minimus*, and *An. dirus* [10]. Female mosquitoes lay their eggs on water surfaces in various habitats, such as salt marshes, lakes or ponds, polluted water retention systems, or any other location where water accumulates. Global warming can alter the ecosystem habitats of various vector species and affect the abundance of vectors that transmit pathogens to humans in various regions [15]. In global climate change, the seasonal and geographical distribution of vector species, especially mosquitoes, are of great importance for persons residing in both rural and urban environments [16]. Therefore, the surveillance and control of mosquitoes are essential for the scientific and effective management of MBIDs and further protection of the civilian community.

Understanding the temporal distribution of mosquitoes can provide an adequate and reliable basis for the development and implementation of mosquito control strategies. Grasping the seasonal characteristics of mosquitoes can provide a reference for mosquito assessment and early warning in the management of public health emergency and MBIDs. Previous literature presented the temporal distribution of mosquitoes in the form of statistical charts that are simple, intuitive, and efficient, but it is difficult to prove in statistics whether differences in peak periods exist among different years or habitats. In recent years, circular statistics have been widely used in the study of the temporal distribution of infectious diseases, providing a comparative analysis of the peak period in different years [17–20]. However, the number of studies on the application of circular statistics in seasonal analysis of mosquitoes is limited. In addition, prior to this study, the mosquito population composition and density in recent years in Qingdao was little explored. Therefore, the purpose of this study is to investigate the application of circular statistics in determining the peak period and a comparison of differences, and to understand adult mosquito densities and composition in different habitats, such as urban dwellings, parks, hospitals, rural dwellings, and livestock sheds, in Qingdao, Shandong Province, China.

## Methods

### Mosquito surveillance

**Fig. 1:**
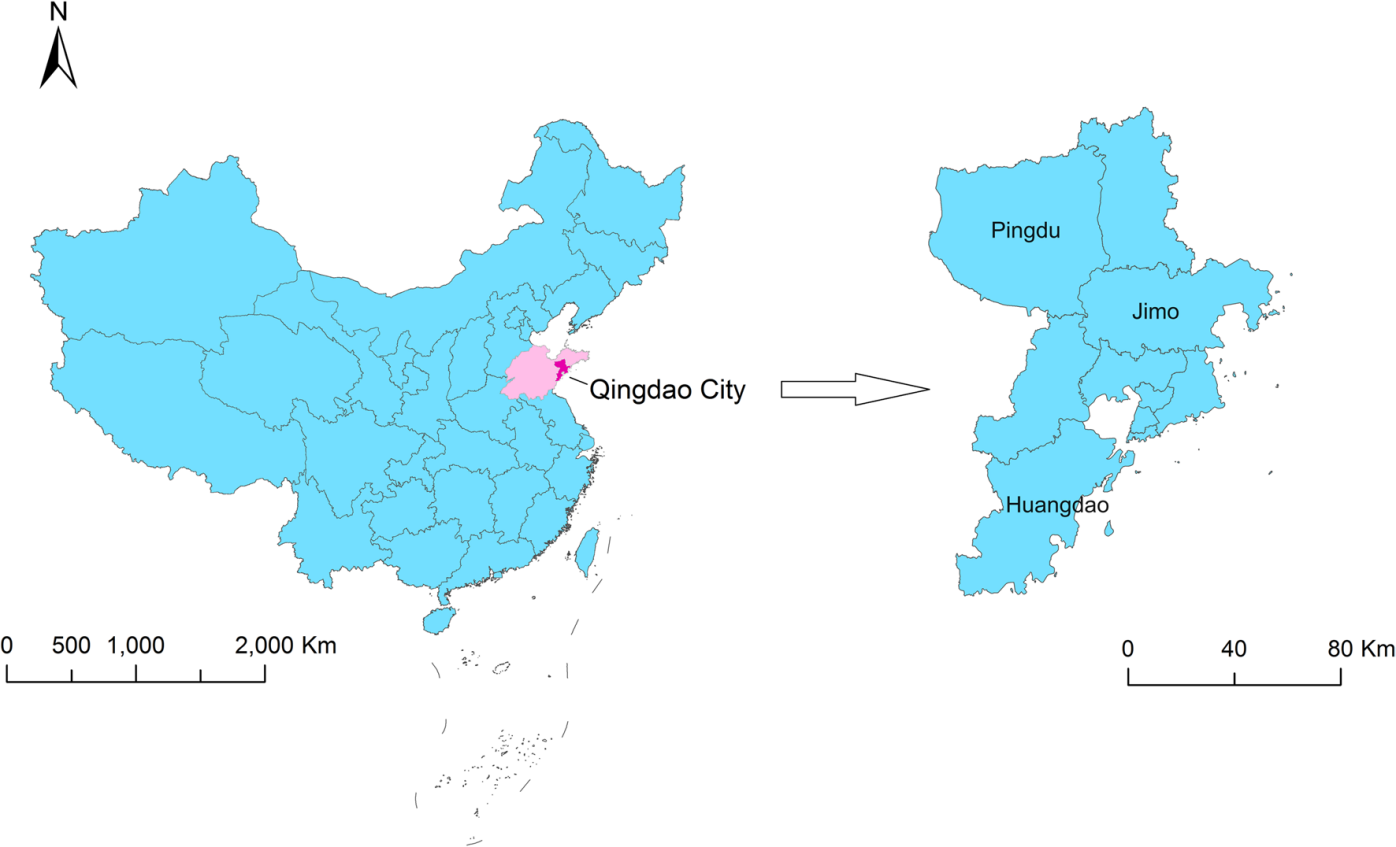
Map of Qingdao City illustrating light traps site locations, Shandong Province, China, 2021–2023

Surveillance of adult mosquito was conducted by light traps from March to November for three years (2021–2023) at three districts in Qingdao, Shandong Province, China. The three chosen districts were Jimo (36° 18–36° 37 North, 120° 07–121° 23 East), Huangdao (35° 35–36° 08 North, 119° 30–120° 18 East), and Pingdu (36° 28–37° 02 North, 119° 31–120° 19 East), which have a temperate monsoon climate (Fig. 1). Jimo, a city of about 1.37 million people, has a land area of approximate 1920.92 km^2^; Huangdao, a city of about 2.61 million people, has a land area of approximate 2128 km^2^; and the region of Pingdu has a population of about 1.18 million people, and its land area is approximately 3175.63 km^2^. Mosquito surveillance was carried out twice a month at 101 sites representing five different habitats, including 17 urban dwelling sites, 16 park sites, 25 hospital sites, 27 rural dwelling sites, and 16 livestock shed sites. Two light traps were placed in each habitat.

The light traps (Lucky Star Environmental Protection Technology Co., Ltd., Wuhan, China) is a tool of mosquito surveillance designated by the Center for Disease Control and Prevention (CDC) in China. Light traps were dependent upon local electrical sources or portable power sources and placed approximately 25–100 m from the nearest external light source. Monitoring devices were hung from columns in the inside of livestock shed or on trees about 1.5 m above the ground. All traps used 6-W near-UV fluorescent light lamps (365 nm) as the attractant. The light traps were operated between the hours of dusk and dawn (18:00–8:00) twice a month. All mosquitoes collected in the trap nets were transferred to local CDC laboratory and frozen to death. All captured adult mosquitoes were morphologically identified with a dissecting microscope (ZEISS, Suzhou, China) according to standard keys [21].

### Circular statistics

The peak period and comparison of differences were evaluated by circular statistics [22]. The months of the year were placed around a circumference of a circle where 1 day is equal to 0.9863°. We took the zero hour of New Year’s Day as the zero angle, and the angle of each month in a year was determined accordingly. The monthly median, the midpoint of the angular interval corresponding to each month, was taken as the middle value of the group and then converted into degrees (i.e., 15.7808° for January; 44.3835° for February). The monthly number of mosquitoes was assigned to the monthly median. Thus, the circular distribution was calculated using the following formula: $X\, = \,\frac{{\sum {f_{i} \cos \alpha_{i} } }}{n}$, $Y\, = \,\frac{{\sum {f_{i} \sin \alpha_{i} } }}{n}$, $\gamma \, = \,\sqrt {X^{2} \, + \,Y^{2} }$, , , $s = \frac{180^\circ }{{\pi \sqrt { - 2\ln \gamma } }}$, where *ƒ*_*i*_ is the monthly data of mosquito, *α*_*i*_ is the monthly median, *n* is the total data of mosquito, *s* is the angular deviation, and $\overline{\alpha }$ represents the mean angle of a sample. The mean angle of circular distribution is used to express the concentrating direction of mosquito activity and provide the peak period of mosquito activity. Rayleigh’s *z* was used to test the significance of the mean angle (*α* = 0.05). *z* was calculated using the following formula:$z=n{\gamma }^{2}$. After obtaining the mean angle of $\overline{\alpha }$, we could use “$\overline{\alpha }$ ± *s*” to estimate the peak period of mosquito activity. 

The differences in peak period of mosquito activity among different years and habitats were determined by the Watson–William test. The Watson–William test is carried out through the following formula: $F=\frac{K(N-k)(\sum {R}_{j}-R)}{(k-1)(N-\sum {R}_{j})}$, where *N* is the total of different sample contents; *k* is the number of compared samples; *R* is calculated by $R={n}_{j}{\gamma }_{j}$ combined with the data from the *k* samples; *R*_*j*_ is the *R* value of different samples considered separately; and *K* is a factor that corrects bias in the *F* calculation. We can determine the value of *K* according to the *γ* value. The critical value of the *F* test can be obtained according to the degree of freedom (*n*_*1*_ = *k* − 1, *n*_*2*_ = *N* − *k*) by looking up the common table of variance.

### Statistical analysis

Mosquito density was calculated as the average number of female mosquitoes per trap per night (females/trap night). All *P* values were two-tailed, and *P* values of less than 0.05 were considered to indicate statistical significance. The software of WPS Excel 2020 (Kingsoft Corp., Beijing, China) and IBM SPSS 27 (IBM Corp., Armonk, NY, USA) was used for all analyses.

## Results

### Mosquito population composition

A total of 14,834 adult mosquitoes comprising five mosquito species from four genera were collected and identified in all habitats during the years 2021–2023. Among five mosquito species, *Culex pipiens pallens* was the dominant species (Tables 1 and 2).

**Table 1:** Mosquito population composition among different years (2021–2023) in Qingdao, Shandong Province, China

Year	Sex	Number (%)					
		*Culex pipiens pallens*	*Culex tritaeniorhynchu*	*Aedes albopictus*	*Anopheles sinensis*	*Armigeres subalbatus*	Total
2021	Female	3567 (96.6)	0 (0.0)	4 (0.1)	48 (1.3)	74 (2.0)	3693 (100.0)
	Male	1506 (95.9)	0 (0.0)	1 (0.1)	4 (0.3)	59 (3.7)	1570 (100.0)
	Unable to identify	66 (100.0)	0 (0.0)	0 (0.0)	0 (0.0)	0 (0.0)	66 (100.0)
	Total	5139 (96.4)	0 (0.0)	5 (0.1)	52 (1.0)	133 (2.5)	5329 (100.0)
2022	Female	1685 (83.5)	1 (0.0)	142 (7.0)	12 (0.6)	179 (8.9)	2019 (100.0)
	Male	3712 (86.3)	0 (0.0)	426 (9.9)	2 (0.1)	160 (3.7)	4300 (100.0)
	Unable to identify	72 (80.0)	0 (0.0)	10 (11.1)	0 (0.0)	8 (8.9)	90 (100.0)
	Total	5469 (85.3)	1 (0.0)	578 (9.0)	14 (0.2)	347 (5.5)	6409 (100.0)
2023	Female	1137 (86.6)	0 (0.0)	80 (6.1)	11 (0.8)	85 (6.5)	1313 (100.0)
	Male	1528 (86.4)	0 (0.0)	179 (10.1)	4 (0.2)	58 (3.3)	1769 (100.0)
	Unable to identify	14 (100.0)	0 (0.0)	0 (0.0)	0 (0.0)	0 (0.0)	14 (100.0)
	Total	2679 (86.5)	0 (0.0)	259 (8.4)	15 (0.5)	143 (4.6)	3096 (100.0)
Total	Female	6389 (91.0)	1 (0.0)	226 (3.2)	71 (1.0)	338 (4.8)	7025 (100.0)
	Male	6746 (88.3)	0 (0.0)	606 (7.9)	10 (0.1)	277 (3.7)	7639 (100.0)
	Unable to identify	152 (89.4)	0 (0.0)	10 (5.9)	0 (0.0)	8 (4.7)	170 (100.0)
	Total	13287 (89.6)	1 (0.0)	842 (5.7)	81 (0.5)	623 (4.2)	14834 (100.0)

**Table 2:** Mosquito population composition among five different habitats in Qingdao, Shandong Province, China, 2021–2023

Habitats	Sex	Number (%)					
		*Culex pipiens pallens*	*Culex tritaeniorhynchu*	*Aedes albopictus*	*Anopheles sinensis*	*Armigeres subalbatus*	Total
Urban dwellings	Female	1114 (93.5)	0 (0.0)	39 (3.3)	12 (1.0)	27 (2.2)	1192 (100.0)
	Male	1281 (90.9)	0 (0.0)	110 (7.8)	2 (0.1)	17 (1.2)	1410 (100.0)
	Unable to identify	26 (96.3)	0 (0.0)	0 (0.0)	0 (0.0)	1 (3.7)	27 (100.0)
	Total	2421 (92.1)	0 (0.0)	149 (5.7)	14 (0.5)	45 (1.7)	2629 (100.0)
Parks	Female	1329 (90.2)	1 (0.1)	52 (3.5)	35 (2.4)	56 (3.8)	1473 (100.0)
	Male	1385 (88.5)	0 (0.0)	144 (9.2)	2 (0.1)	34 (2.2)	1565 (100.0)
	Unable to identify	34 (91.9)	0 (0.0)	2 (5.4)	0 (0.0)	1 (2.7)	37 (100.0)
	Total	2748 (89.4)	1 (0.0)	198 (6.4)	37 (1.2)	91 (3.0)	3075 (100.0)
Hospitals	Female	1108 (94.7)	0 (0.0)	28 (2.4)	0 (0.0)	34 (2.9)	1170 (100.0)
	Male	1183 (89.1)	0 (0.0)	113 (8.5)	1 (0.1)	30 (2.3)	1327 (100.0)
	Unable to identify	33 (97.1)	0 (0.0)	1 (2.9)	0 (0.0)	0 (0.0)	34 (100.0)
	Total	2324 (91.8)	0 (0.0)	142 (5.6)	1 (0.0)	64 (2.6)	2531 (100.0)
Rural dwellings	Female	1131 (89.3)	0 (0.0)	54 (4.3)	6 (0.5)	76 (5.9)	1267 (100.0)
	Male	1318 (88.2)	0 (0.0)	119 (8.0)	0 (0.0)	58 (3.8)	1495 (100.0)
	Unable to identify	25 (75.8)	0 (0.0)	4 (12.1)	0 (0.0)	4 (12.1)	33 (100.0)
	Total	2474 (88.5)	0 (0.0)	177 (6.3)	6 (0.2)	138 (5.0)	2795 (100.0)
Livestock sheds	Female	1707 (88.8)	0 (0.0)	53 (2.8)	18 (0.9)	145 (7.5)	1923 (100.0)
	Male	1579 (85.7)	0 (0.0)	120 (6.5)	5 (0.3)	138 (7.5)	1842 (100.0)
	Unable to identify	34 (87.2)	0 (0.0)	3 (7.7)	0 (0.0)	2 (5.1)	39 (100.0)
	Total	3320 (87.3)	0 (0.0)	176 (4.6)	23 (0.6)	285 (7.5)	3804 (100.0)

### Mosquito population density

**Fig. 2:**
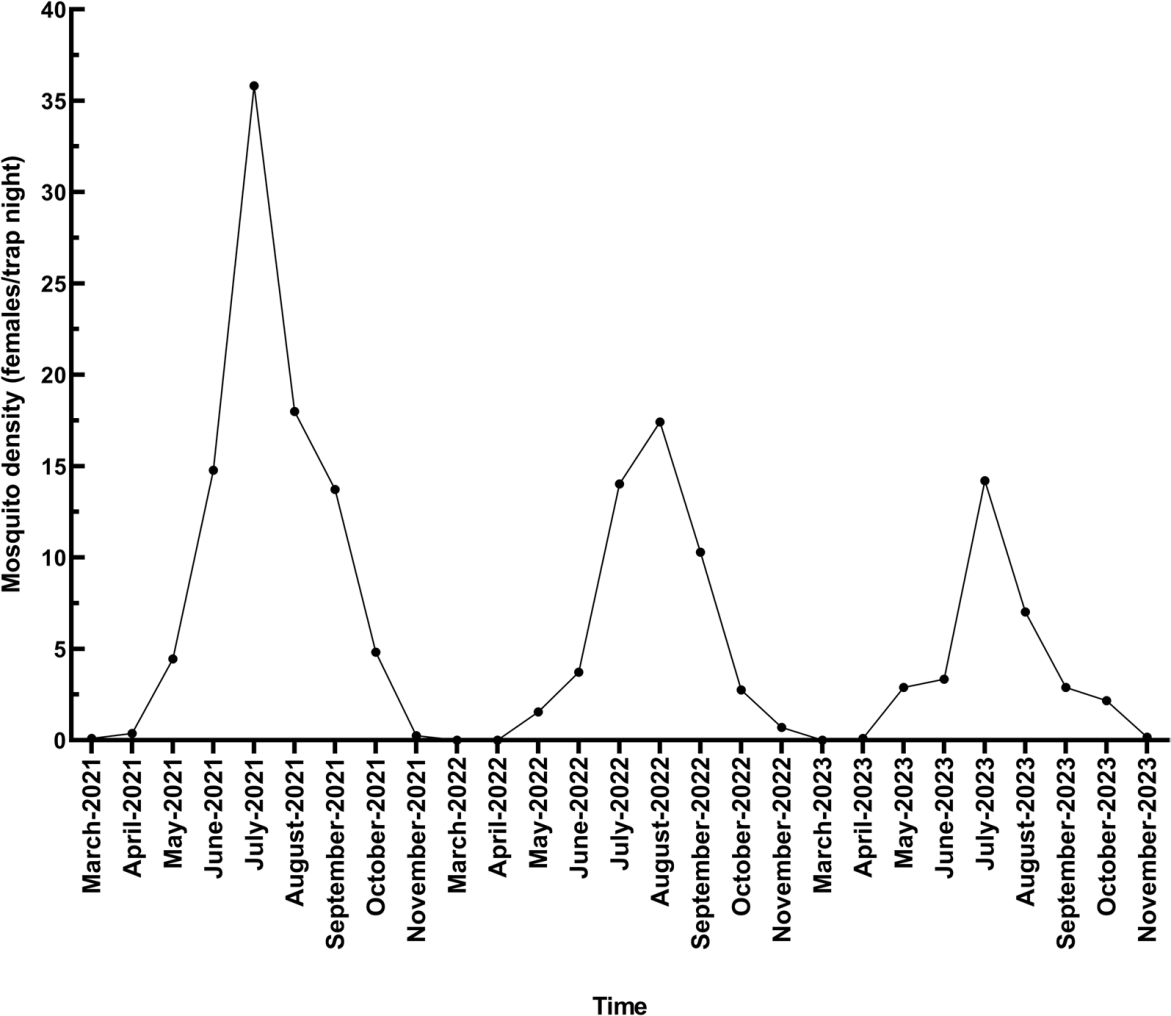
Dynamics of all mosquitoes from March to November among 3 years (2021–2023) in Qingdao, Shandong Province, China

Figure 2 shows the dynamics of all mosquitoes from March to November in Qingdao City, 2021–2023. All mosquitoes were captured in 1080 trap nights from March to November for 3 years. The mean mosquito density (females/trap night) for the trapping period was 10.3 in 2021, 5.6 in 2022, and 3.6 in 2023, and no statistically significant difference was found in mosquito density among different years (*H* = 1.96, d.f. 2, *P* = 0.376).

**Fig. 3:**
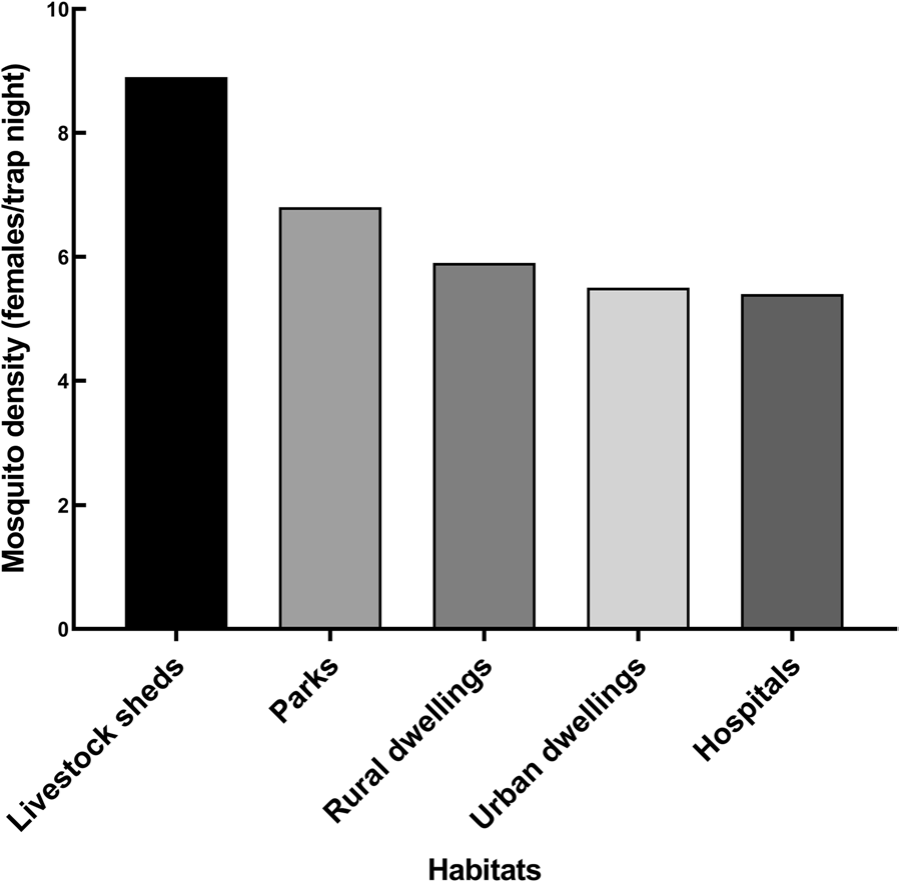
Mosquito density among five different habitats in Qingdao, Shandong Province, China, 2021–2023

Among five different habitats, the highest mosquito density was 8.9 in livestock sheds, followed by 6.8 in parks, 5.9 in rural dwellings, 5.5 in urban dwellings, and 5.4 in hospitals (Fig. 3), and there was no statistically significant difference in mosquito density among different habitats (*H* = 0.45, d.f. 4, *P* = 0.978).

### Peak period of mosquito activity and comparison of differences among different years

Table 3 presents the peak period of mosquito activity in 2021–2023. The seasonal fluctuation peak among 3 years differs significantly (*F*_(2,7022)_ = 119.17, *P* < 0.05).

**Table 3:** Circular distribution of all mosquitoes activity in 2021–2023

	*γ*	$\bar\alpha$	*Z*	*P* value	*S*	Peak period
2021	0.804	205.382	2386.949	< 0.01	37.852	19 June to 4 September
2022	0.828	219.692	1383.062	< 0.01	35.242	7 July to 16 September
2023	0.799	203.286	837.895	< 0.01	38.401	17 June to 3 September
2021–2023	0.804	209.185	4542.245	< 0.01	37.836	23 June to 8 September

### Peak period of mosquito activity and comparison of differences among different habitats

Table 4 presents the peak period of mosquito activity in the urban dwellings, parks, hospitals, rural dwellings, and livestock sheds. The seasonal fluctuation peak among five different habitats did not differ significantly (*F*_*(4,7020)*_ = −159.09, *P* > 0.05).

**Table 4:** Circular distribution of all mosquitoes activity in five different habitats

	*γ*	$\bar\alpha$	*Z*	*P* value	*S*	Peak period
Urban dwellings	0.796	204.764	754.544	< 0.01	38.746	18 June to 4 September
Parks	0.812	211.626	970.103	< 0.01	37.029	27 June to 10 September
Hospitals	0.801	202.646	750.865	< 0.01	38.159	16 June to 2 September
Rural dwellings	0.825	210.648	862.996	< 0.01	35.506	27 June to 7 September
Livestock sheds	0.799	213.001	1226.655	< 0.01	38.419	27 June to 12 September
Integration	0.804	209.185	4542.245	< 0.01	37.836	23 June to 8 September

## Discussion

The mosquito is a vector with an enormous public health burden, causing millions of deaths and hundreds of millions of disease cases annually [23, 24]. Recently, global warming has intensified concerns regarding MBIDs owing to the expanding distribution of arboviruses transmitted by mosquitoes in recent years. Furthermore, global warming may shorten vector life cycles, facilitate larger mosquito populations, and enhance disease transmission [25]. In fact, China is experiencing risks of reemergence of previously eradicated vector diseases and spread of exotic MBIDs. Therefore, continuous, systematic, and seasonal surveillance of mosquitoes is required because it is technically difficult to prevent the spread of MBIDs from foreign countries through quarantine alone. Considering the enormous public health challenge posed by mosquito infestation, we conducted a 3-year surveillance of mosquito populations in different habitats, including urban dwellings, parks, hospitals, rural dwellings, and livestock sheds, in Qingdao, Shandong Province, China.

According to the requirements of the national standard for mosquito surveillance in China and the National Vector Surveillance Implementation Plan (2016) formulated by the China CDC, the number of female mosquitoes captured in surveillance was used to calculate mosquito density. Therefore, males collected were excluded from the seasonal analysis in this study. In addition, to maturate the fertilized eggs, female mosquitoes suck human blood by biting and disseminating the virus between the human host and mosquito vector. Thus, it is of public health significance to study female mosquito density and seasonal fluctuation. The surveillance results exhibited a decreasing trend in mosquito density from 2021 to 2023, similar to the City of Liaocheng in Shandong Province in China [26]. A reasonable explanation for this phenomenon may be associated with the reduction of mosquito breeding habitats due to the intensification of environmental health. Among five different habitats, the density of mosquitoes in the livestock sheds was highest, followed by parks, rural dwellings, urban dwellings, and hospitals. Several explanations were considered for these findings. On the one hand, the high density of mosquitoes in the livestock sheds may be related to poor sanitation conditions and more sewage ditches in this habitat. On the other hand, all kinds of animals in livestock sheds can provide a sufficient blood source for mosquitoes. Additionally, the low density of mosquitoes in the urban dwellings and hospitals may be related to the continuous Patriotic Health Campaign (PHC) organized by the government in an urban region in Qingdao City. PHC promotes the appearance, environment sanitation, and public health including vector management, especially mosquito management, of cities in Qingdao.

In terms of mosquito composition, the present study revealed that the proportion of *Cx. pipiens pallens* was much higher than that of other mosquito species. Light traps utilized in a specific habitat at night had a good effect on attracting *Cx. pipiens pallens* mainly active at night [27]. Moreover, *Cx. pipiens pallens* is the main blood-sucking mosquito species in the northern area of China, and it mainly breeds in moderately polluted water near human settlements where current surveillance was carried out, such as ditches, underground garages, septic tanks, and stink ditches [28]. Despite the lower proportion of other mosquitoes in this study, the risks posed by other mosquito species still need to be investigated. For example, malaria is an infectious disease caused by *Plasmodium* parasites, transmitted by *An. sinensis*, *An. lesteri*, *An. minimus*, and *An. dirus* in China. In addition, *Ae. albopictus* is a major disease vector that can transmit several important arboviruses, including chikungunya, dengue, yellow fever, and Zika viruses [29], and this is one of the most common mosquito species in China [30]. Therefore, further investigations on these mosquito species are advocated.

With respect to the temporal distribution of the mosquito population, statistical charts and circular statistics are used jointly in seasonal analysis. Mosquito activity began to increase around May and occurred until October in Qingdao, and large numbers of mosquitoes were collected between June and September. Meanwhile, the mean angle of circular distribution was adopted to determine the definite time focus, revealing that the seasonal peak period of mosquito activity fell in the months from June to September. With circular statistics, one can not only determine the peak period but also compare the differences in statistics among different years and habitats. Furthermore, circular statistics make up for the deficiency of statistical charts, providing a theoretical basis for the seasonal analysis of mosquito. Although the peak of mosquito density among different years differs in statistics, the peak period was concentrated in the seasons of summer and autumn characterized by hot, wet, and rainy conditions suitable for mosquito breeding. Nevertheless, no significant difference in peak period among different habitats was discovered in this study.

This study has several policy implications. MBIDs continue to be a threat to people of the world, especially in China. The Chinese government has taken various measures to reduce the risk of MBIDs, including the Patriotic Health Campaign and creation of “National Sanitary City,” and take initiative in mosquito vector management [31]. However, MBIDs remain an important public health concern since the distribution is showing a tendency to expand and the incidence of many MBIDs still remains at a relatively high level. It is widely accepted that mosquito management is essential for the prevention and control of MBIDs [32, 33]. Therefore, understanding the seasonal distribution of mosquitoes can provide an adequate and reliable basis for the development and implementation of mosquito control strategies. Moreover, it is important for government to adopt a multisectoral cooperation approach aimed at controlling mosquitoes and managing breeding habitats to reduce the distribution and incidence of MBIDs.

## Conclusions

Circular statistics could be effectively combined with statistical charts to elucidate the peak period of mosquitoes and determine the differences in statistics among different years or habitats. Furthermore, these results will provide valuable information for mosquito control and public health management.

## Data Availability

All data generated or analyzed during this study are included in this published article.

## Abbreviations

VBIDs: Vector-borne infectious diseases
MBIDs: Mosquito-borne infectious diseases
CDC: Centers for Disease Control and Prevention
PHC: Patriotic Health Campaign
